# Waveform Design for the Integrated Sensing, Communication, and Simultaneous Wireless Information and Power Transfer System

**DOI:** 10.3390/s24134129

**Published:** 2024-06-25

**Authors:** Qilong Miao, Weimin Shi, Chenfei Xie, Yong Gao, Lu Chen

**Affiliations:** 1 School of Information and Communication Engineering, University of Electronic Science and Technology of China, Chengdu 611731, China; 201911012021@std.uestc.edu.cn; 2 School of Microelectronics and Communication Engineering, Chongqing University, Chongqing 400044, China; 3 National Key Laboratory of Wireless Communications, University of Electronic Science and Technology of China, Chengdu 611731, China; chenff93625@163.com; 4 School of Electronic Science and Engineering, University of Electronic Science and Technology of China, Chengdu 611731, China; gaoyong@uestc.edu.cn; 5 School of Aeronautics and Astronautics, University of Electronic Science and Technology of China, Chengdu 611731, China; lchen@std.uestc.edu.cn

**Keywords:** simultaneous wireless information and power transfer (SWIPT), integrated sensing and communication system (ISACS), orthogonal time frequency space (OTFS), waveform design, beamforming, semidefinite relaxation (SDR), matched filter (MF)

## Abstract

Next-generation communication systems demand the integration of sensing, communication, and power transfer (PT) capabilities, requiring high spectral efficiency, energy efficiency, and low cost while also necessitating robustness in high-speed scenarios. Integrated sensing and communication systems (ISACSs) exhibit the ability to simultaneously perform communication and sensing tasks using a single RF signal, while simultaneous wireless information and power transfer (SWIPT) systems can handle simultaneous information and energy transmission, and orthogonal time frequency space (OTFS) signals are adept at handling high Doppler scenarios. Combining the advantages of these three technologies, a novel cyclic prefix (CP) OTFS-based integrated simultaneous wireless sensing, communication, and power transfer system (ISWSCPTS) framework is proposed in this work. Within the ISWSCPTS, the CP-OTFS matched filter (MF)-based target detection and parameter estimation (MF-TDaPE) algorithm is proposed to endow the system with sensing capabilities. To enhance the system’s sensing capability, a waveform design algorithm based on CP-OTFS ambiguity function shaping (AFS) is proposed, which is solved by an iterative method. Furthermore, to maximize the system’s sensing performance under communication and PT quality of service (QoS) constraints, a semidefinite relaxation (SDR) beamforming design (SDR-BD) algorithm is proposed, which is solved using through the SDR technique. The simulation results demonstrate that the ISWSCPTS exhibits stronger parameter estimation performance in high-speed scenarios compared to orthogonal frequency division multiplexing (OFDM), the waveform designed by CP-OTFS AFS demonstrates superior interference resilience, and the beamforming designed by SDR-BD strikes a balance in the overall performance of the ISWSCPTS.

## 1. Introduction

Next-generation communication systems require the integration of additional functionalities to enhance system spectral efficiency, energy efficiency, and reduce system costs. Integrated sensing and communication systems (ISACSs), which converge sensing and communication functionalities within a unified framework, have gained significant traction in recent years [1,2,3,4]. By co-locating sensing and communication tasks and sharing common resources such as antennas and signal processing algorithms, ISACS promise enhanced performance, reduced latency, and improved resource utilization compared to traditional separate implementations. However, the functionalities of ISACSs still do not fully leverage the potential of wireless radio frequency signals, such as power transfer (PT).

Certain modern wireless applications impose energy transfer requirements on ISACSs. One such example is a battlefield unmanned aerial vehicle (UAV). In combat environments, UAVs operating under constrained resources need to simultaneously accomplish target perception, transmit information, and enhance endurance. One approach to addressing this challenge is the efficient and rational allocation of power in ISACSs to improve energy efficiency. Some existing works have studied power allocation problems in ISACSs. Another approach is equipping ISACS with energy-harvesting devices to collect energy from received radio frequency (RF) signals, thereby extending the system’s operational lifespan. Regarding the latter, simultaneous wireless information and power transfer (SWIPT), first proposed in [5], has garnered significant attention in recent years [6,7,8]. The core of SWIPT is to use a single signal as a carrier for both information transmission and energy transfer. Communication nodes extract information from the received signal, while energy-harvesting nodes collect energy from it. Clearly inspired by ISACS, SWIPT still has the potential to expand perception capabilities and further improve system efficiency.

In ISACS, the transmitted waveform plays a crucial role in determining system performance. Waveforms based on orthogonal frequency division multiplexing (OFDM), extensively used in fourth-generation (4G) and emerging fifth-generation (5G) cellular systems as well as WiFi networks, are a promising choice due to their simple processing framework and high processing gain. However, OFDM encounters challenges in high-mobility scenarios. The OFDM waveform suffers from severe inter-carrier interference (ICI), exacerbated by varying normalized Doppler shifts between the highest and lowest subcarriers. Consequently, achieving synchronization becomes challenging. Similar to communication systems, the performance of OFDM-based waveforms sharply deteriorates in high-speed mobile scenarios [9]. Next-generation communication systems also demand robustness in high-Doppler time-varying scenarios, as is required for ISACS. Orthogonal time frequency space (OTFS), a modulation scheme that modulates/demodulates information in the Doppler-delay (DD) domain, has been proposed in recent years [10,11,12]. OTFS represents high-Doppler time-varying channels that are sparse in the DD domain. Previous studies have shown that OTFS offers significant communication performance improvements compared to OFDM in high-speed time-varying scenarios [11,12]. OTFS is also utilized in sensing systems and demonstrates excellent performance compared to OFDM [9,13,14]. Due to the aforementioned advantages, the cyclic prefix OTFS (CP-OTFS) is adopted as the fundamental waveform in this work.

Currently, there is relatively limited research on the integration of ISACS with SWIPT. In [15], the Cramer–Rao lower bound (CRLB) of the estimation of the targets’ angle is employed as the optimization objective, and the beamforming is designed with communication and power transfer quality of service (QoS) constraints in mind. We analyze the shortcomings of existing technologies, as depicted in Table 1, to clarify the motivation, innovation, and significance of this work.

In this work, we propose a framework for a cyclic prefix (CP)-OTFS-based integrated simultaneous wireless sensing, communication, and power transfer system (ISWSCPTS), which incorporates both a search mode (SM) and joint work mode (JWM). This framework integrates the advantages of ISACS and SWIPT, enabling the simultaneous perception of targets, information transmission, and energy transfer capabilities. The main contributions of this work are as follows:

- The framework of ISWSCPTS is proposed. This framework comprises two operational modes: SM and JWM. To enhance sensing capabilities for both SM and JWM, the CP-OTFS matched filter (MF)-based target detection and parameter estimation (MF-TDaPE) algorithm is proposed. This algorithm exhibits superior performance to OFDM in high-speed scenarios.
- A CP-OTFS ambiguity function (AF) shaping (AFS) waveform design algorithm is proposed. Firstly, a novel DD domain AF for CP-OTFS is proposed. Subsequently, aiming to minimize the integrated sidelobe level (ISL) of the proposed AF while adhering to the QoS for communication, PT, and constant modulus constraints, a non-convex CP-OTFS waveform design optimization problem is formulated. This problem is then solved through an iterative algorithm to obtain waveforms with superior AF characteristics and interference resilience.
- The semidefinite relaxation (SDR) beamforming design (SDR-BD) algorithm tailored for ISWSCPTS is proposed. Initially, a non-convex optimization problem is formulated with sensing QoS as the optimization objective and communication and PT QoS as constraints. Subsequently, the SDR technique is employed to solve this problem. The designed waveform optimizes perceptual capability while ensuring communication and PT performance.

The structure of the paper is outlined as follows: Section 2.1 introduces the principles of CP-OTFS. Section 2.2 elaborates on the framework principles, waveform design, and beamforming design. Section 3 presents simulation results and corresponding discussions. Finally, Section 4 concludes with a summary and outlines future work prospects.

## 2. Materials and Methods

**Figure 1:**
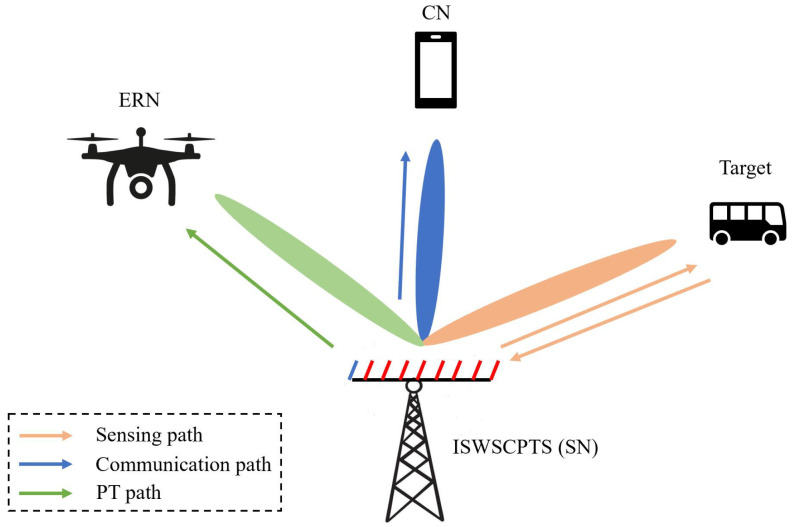
The structure of ISWSCPTS.

Consider an CP-OTFS-based ISWSCPTS equipped with a uniform linear array (ULA) with $N_{t}$ transmitting elements and a single receiving antenna to sense a single target, deliver information to a single communication node (CN), and transfer energy to a single energy receiving node (ERN), as shown in Figure 1. The ISWSCPTS transmits a single information-carrying CP-OTFS signal. Through this multifunctional signal, the ISWSCPTS receives a target echo to complete the perception, the CN receives the signal to accomplish the information collection, and the ERN receives the signal to complete the energy-harvesting. The ISWSCPTS is also referred to as a sensing node (SN) due to its function of sensing. We detail the system model of ISWSCPTS starting from the CP-OTFS model.

### 2.1. The CP-OTFS Model

#### 2.1.1. Basic Concepts of CP-OTFS

The time–frequency (TF) plane is discretized to a N×M grid, as follows:

(1) Δ=(nT,mΔf),n=0,…,N−1,m=0,…,M−1

where *T* and Δf=1/T are sampling intervals of time and frequency axes, respectively.

The modulated TF samples χ[n,m] are transmitted over an OTFS frame with duration $\hat{T}=NT$ and occupy a bandwidth B=MΔf. The sampling frequency $f_{s}=B$.

The DD plane is discretized to an N×M lattice as follows [12]:

(2) $$\Xi =(\frac{k}{NT},\frac{l}{M\Delta f}),k=0,\ldots ,N-1,l=0,\ldots ,M-1.$$

#### 2.1.2. Input–Output Relationship of CP-OTFS-Based ISWSCPTS

*A. The transmitter model*

**Figure 2:**
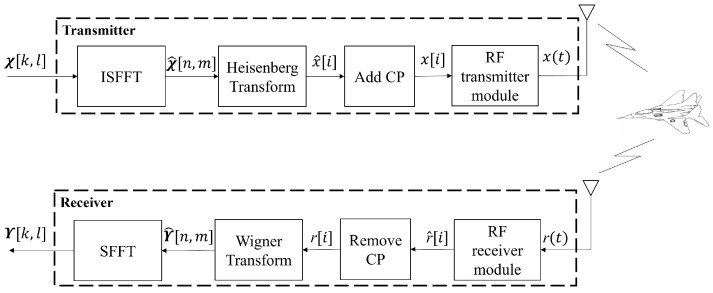
The structure of CP-OTFS.

The CP-OTFS-based system is depicted in Figure 2. The symbols χ[k,l] in the DD domain are first transformed into symbols $\hat{\chi }\left[n,m\right]$ in TF domain by the inverse symplectic finite Fourier transform (ISFFT). Then, the Heisenberg transform is applied to $\hat{\chi }$ to generate time domain discrete signal $\hat{x}\left[i\right],i=0,\ldots ,NM-1$. The expression of $\hat{x}\left[i\right]$ is

(3) $$\begin{array}{cc} \hat{x}\left[i\right]= & \sum_{n^{\prime }}^{N-1}\sum_{m^{\prime }}^{M-1}\sum_{k^{\prime }}^{N-1}\sum_{l^{\prime }}^{M-1}\chi \left[k^{\prime },l^{\prime }\right]\\ & \exp\left\{j2\pi \left(\frac{k^{\prime }n^{\prime }}{N}-\frac{l^{\prime }m^{\prime }}{M}+\frac{m^{\prime }i}{M}\right)\right\}g_{tx}\left[i-n^{\prime }M\right], \end{array}$$

where the $g_{tx}\left[\iota \right]$ is the transmitting rectangular pulse and its expression is

(4) $$g_{tx}\left[\iota \right]=\left\{\begin{array}{c} 1,0\leq \iota \leq M-1\\ 0,otherwise \end{array}\right.$$

Next, the discrete CP-OTFS transmitting signal x[i] is constructed by adding a CP of length *M* to x[i]. Finally, x[i] is converted to radio frequency (RF) signal x(t) by an RF transmitter module.

*B. The channel model*

In the application scenarios of ISWSCPTS, the perception target, communication channel, and energy transmission channel can all be modeled using a unified framework based on the DD channel (DDC) model. The traditional TD channel model assumes that the channel impulse response (CIR) is time-invariant. However, with increasing mobility and carrier frequency, this time-invariant assumption may no longer hold. Therefore, new channel models need to consider the CIR in both the TD and DD dimensions. As pointed out in  [10], DDC exhibits beneficial features such as separability, stability, compactness, and possible sparsity. Hence, this paper adopts DDC.

Assume there are *P* DDCs which can be expressed as [9]

(5) $$\rho \left(\tau ,f_{d}\right)=\sum_{p=1}^{P}\alpha_{p}\delta \left(\tau -\tau_{p}\right)\delta \left(f_{d}-f_{d_{p}}\right),$$

where $\alpha_{p}$ is the channel gain coefficient, $f_{d_{p}}$ is the Doppler, and $\tau_{p}$ is the delay. Let $\alpha ={\left[\alpha_{1},\ldots ,\alpha_{p}\right]}^{T}$.

In this work, we make the following assumptions:

(6) $$\begin{array}{cc} \; & \tau_{p}=l_{p}/M\Delta f,\;l_{p}=0,1,\ldots ,M-1\\ \; & f_{d_{p}}={\left(k_{p}\right)}_{N}/NT,\;k_{p}=0,1,\ldots ,N-1 \end{array}$$

where $l_{p}$ and $k_{p}$ are integers.

Thus, the discrete form of $\rho (\tau ,f_{d})$ can be expressed as

(7) $$\rho \left[k,l\right]=\sum_{p=1}^{P}\alpha_{p}\delta \left[k-{\left(k_{p}\right)}_{N}\right]\delta \left[l-l_{p}\right]$$

where

(8) $${\left(k_{p}\right)}_{N}=\left\{\begin{array}{c} k_{p},k_{p}\leq N/2\\ N-k_{p},otherwise \end{array}\right.$$

*C. The receiver model*

The received signal r(t) is transformed into the discrete-time signal r[i],i=0,…,NM−1, by the RF receiver module and by removing the CP. r[i] can be expressed as

(9) $$r\left[i\right]=\sum_{p=1}^{P}\alpha_{p}x\left[{\left[i-l_{p}\right]}_{NM}\right]\exp\left\{j2\pi f_{d_{p}}\left(\frac{iT}{M}+T\right)\right\}+\hat{w}\left[i\right]$$

where ${\left[•\right]}_{NM}$ denotes modulo NM operation, and $\hat{w}\left[i\right]$ is the additive Gaussian white noise (AWGN).

r[i] is converted to received symbols $\hat{\Upsilon }\left[n,m\right]$ in the TF domain through the Wigner transform. $\hat{\Upsilon }\left[n,m\right]$ can be expressed as

(10) $$\hat{\Upsilon }\left[n,m\right]=\sum_{i=0}^{NM-1}r\left[i\right]g_{rx}^{*}\left[i-nM\right]\exp\left(j2\pi \frac{mi}{M}\right)$$

where $g_{rx}\left[\iota \right]$ is the receiving rectangular pulse, and its expression is

(11) $$g_{rx}\left[\iota \right]=\left\{\begin{array}{c} 1,0\leq \iota \leq M-1\\ 0,otherwise \end{array}\right.$$

Finally, $\hat{\Upsilon }\left[n,m\right]$ is converted into the received symbols Υ[k,l] in the DD domain through the symplectic finite Fourier transform (SFFT). Υ[k,l] can be expressed as

(12) $$\Upsilon \left[k,l\right]=\sum_{n=0}^{N-1}\sum_{m=0}^{M-1}\hat{\Upsilon }\left[n,m\right]\exp\left\{-j2\pi \left(\frac{kn}{N}-\frac{lm}{M}\right)\right\}+W\left[k,l\right]$$

where W is the AWGN matrix.

After laborious derivations, (12) can be recast as

(13) $$\Upsilon \left[k,l\right]=\sum_{k^{\prime }=0}^{N-1}\sum_{l^{\prime }=0}^{M-1}\chi \left[k^{\prime },l^{\prime }\right]H_{k,l}\left[k^{\prime },l^{\prime }\right]+W\left[k,l\right]$$

where the expression of $H_{k,l}\left[k^{\prime },l^{\prime }\right]$ is as follows:

(14) $$H_{k,l}\left[k^{\prime },l^{\prime }\right]=\frac{1}{NM^{2}}\left(H_{ISI}+H_{ICI}\right)\exp\left(j2\pi f_{d}T\right)$$

In (14), $H_{ISI}$ and $H_{ICI}$ are expressed as follows:

(15) $$\begin{array}{cc} H_{ISI}= & \sum_{n=0}^{N-1}\exp\left(j2\pi \frac{n}{N}\left(k^{\prime }-k+f_{d}TN\right)\right)\\ & \sum_{m=0}^{M-1}\sum_{m^{\prime }}^{M-1}\exp\left(\frac{-j2\pi }{M}\left(l^{\prime }m^{\prime }-lm+l_{p}m^{\prime }\right)\right)\\ & \sum_{i=l_{p}}^{M-1}\exp\left(j2\pi \frac{i}{M}\left(m^{\prime }-m+f_{d}T\right)\right) \end{array}$$

(16) $$\begin{array}{cc} H_{ICI}= & \exp\left(-j2\pi \frac{k^{\prime }}{N}\right)\sum_{n=0}^{N-1}\exp\left(j2\pi \frac{n}{N}\left(k^{\prime }-k+f_{d}TN\right)\right)\\ & \sum_{m=0}^{M-1}\sum_{m^{\prime }}^{M-1}\exp\left(\frac{-j2\pi }{M}\left(l^{\prime }m^{\prime }-lm+l_{p}m^{\prime }\right)\right)\\ & \sum_{i=0}^{l_{p}-1}\exp\left(j2\pi \frac{i}{M}\left(m^{\prime }-m+f_{d}T\right)\right) \end{array}$$

According to (13), each receiving symbol is the result of summing all input symbols weighted by $H_{k,l}\left[k^{\prime },l^{\prime }\right]$. Clearly, $H_{k,l}\left[k^{\prime },l^{\prime }\right]$ is related to variables *l*, *l*, $k^{\prime }$, and $l^{\prime }$. The magnitude of the response of $H_{k,l}\left[k^{\prime },l^{\prime }\right]$ is as follows:

(17) $$|H_{k,l}\left[k^{\prime },l^{\prime }\right]|=\left\{\begin{array}{c} 1,\;k^{\prime }={\left[k-k_{p}\right]}_{N},l^{\prime }={\left[l-l_{p}\right]}_{M}\\ 0,\;otherwise \end{array}\right.$$

Therefore, the summation operation in (13) can be removed, and it can be simplified as follows:

(18) $$\Upsilon \left[k,l\right]=\sum_{p=1}^{P}\alpha_{p}\chi \left[{\left[k-k_{p}\right]}_{N},{\left[l-l_{p}\right]}_{M}\right]H_{k,l}\left[k_{p},l_{p}\right]+W\left[k,l\right],$$

where $H_{k,l}\left[k_{p},l_{p}\right]$ is defined in (21), the definitions of $H_{1}$ and $H_{2}$ are (19) and (20), respectively. Note that (19)–(21) are exact expressions without any approximation.


(19) $$\begin{array}{c} H_{1}=N\sum_{m=0}^{M-1}\sum_{m^{\prime }=0}^{M-1}\exp\left\{-j2\pi \frac{l(m^{\prime }-m)}{M}\right\}\sum_{i^{\prime }=l_{p}}^{M-1}\exp\left\{j2\pi \frac{\left(m^{\prime }-m+f_{d_{p}}T\right)i^{\prime }}{M}\right\} \end{array}$$

(20) $$\begin{array}{c} H_{2}=N\exp\left(-j2\pi \frac{{\left[k-k_{p}\right]}_{N}}{N}\right)\sum_{m=0}^{M-1}\sum_{m^{\prime }=0}^{M-1}\exp\left\{-j2\pi \frac{l(m^{\prime }-m)}{M}\right\}\sum_{i^{\prime }=0}^{l_{p}-1}\exp\left\{j2\pi \frac{\left(m^{\prime }-m+f_{d_{p}}T\right)i^{\prime }}{M}\right\} \end{array}$$

According to (3)–(12), after tedious derivations, Υ[k,l] can be expressed as

(21) $$H_{k,l}\left[k_{p},l_{p}\right]=\frac{1}{NM^{2}}\left(H_{1}+H_{2}\right)\exp\left(j2\pi f_{d_{p}}T\right)$$

### 2.2. Framework of CP-OTFS-Based ISWSCPTS

ISWSCPTS encompasses two work modes: the SM and JWM for perception, communication, and PT. The following provides a detailed description of these two modes.

#### 2.2.1. The SM of ISWSCPTS

In the SM, the ISWSCPTS lacks a priori information on DDCs for the target, communication, and power transfer, necessitating the estimation of these parameters. In this mode, the ISWSCPTS scans the region of interest in a phased-array manner to acquire the states of DDCs from various angles.

The TF domain-received target echo whose angle is the same as the searching direction $R_{SN}\in C^{NM\times 1}$ can be expressed as

(22) $$R_{SN}=\sqrt{P_{t}}\alpha a_{t}^{H}\left(\theta_{tar}\right)\eta s_{SM}+w_{SN},$$

where $P_{t}$ is the transmitting power, α is the target scattering coefficient (TSC), $s_{SM}$ is the TF domain transmitting signal in the search mode, $w_{SN}\in C^{NM\times 1}$ is the AWGN matrix with the variance of each entry being $\sigma_{SN}^{2}$, $\theta_{tar}$ is the angle of the target, $a_{t}\left(\theta_{tar}\right)\in C^{N_{t}\times 1}$ is the steering vector, and $a_{t}\left(\theta_{tar}\right)$ can be expressed as

(23) $$\begin{array}{cc} a_{t}\left(\theta_{tar}\right)= & [1,\exp\left(-j2\pi \frac{d\sin(\theta_{tar})}{\lambda_{c}}\right),\exp\left(-j2\pi 2\frac{d\sin(\theta_{tar})}{\lambda_{c}}\right),\ldots ,\\ \; & \exp\left(-j2\pi \left(N_{t}-1\right)\frac{d\sin(\theta_{tar})}{\lambda_{c}}\right){]}^{T}, \end{array}$$

where *d* is the antenna spacing, $\lambda_{c}$ represents the wavelength.

In the phased-array manner, $\eta =a_{t}\left(\theta_{tar}\right)$; thus, (22) can be reformulated as

(24) $$R_{SN}=\sqrt{P_{t}}\alpha N_{t}s_{SM}+w_{SN},$$

According to (18), the vector form of the DD domain echo $y_{SN}\in C^{NM\times 1}$ can be expressed as

(25) $$y_{SN}=\sqrt{P_{t}}\alpha N_{t}\left(h_{SN}⊙{\hat{x}}_{SN}\right)+w_{SN},$$

where $h_{SN}=\mathrm{vec}\left(H_{SN}\right)$, ${\hat{x}}_{SN}=\mathrm{vec}\left({\hat{X}}_{SN}\right)$, vec(•) vectorizes the matrix along the row direction. For a target with delay tap $l_{tar}$ and Doppler tap $k_{tar}$, the (k,l)-th entry of $H_{SN}$ can be expressed as

(26) $$H_{SN}\left[k,l\right]=H_{k,l}\left[k_{tar},l_{tar}\right].$$

The (k,l)-th entry of $X_{SN}$ can be expressed as

(27) $${\hat{X}}_{SN}\left[k,l\right]=\chi \left[{\left[k-k_{tar}\right]}_{N},{\left[l-l_{tar}\right]}_{M}\right].$$

The MF algorithm proposed in Reference [9] represents a state-of-the-art approach for OTFS-based target detection and parameter estimation. In this work, we offer a re-engineered version of the MF algorithm, employing an equivalent yet distinct methodology. Note that (25) can be reformulated as

(28) $$y_{SN}=\sqrt{P_{t}}\alpha N_{t}\Lambda_{SN}\left(k_{tar},l_{tar}\right)P_{SN}\left(k_{tar},l_{tar}\right)x_{SM}+w_{SN},$$

where $x_{SM}=\mathrm{vec}\left(\chi \right)$, $\Lambda_{SN}\left(k_{tar},l_{tar}\right)=\mathrm{diag}\left(h_{SN}\right)$, $P_{SN}\left(k_{tar},l_{tar}\right)$ is the permutation matrix. The (i,j)-th entry of $P_{SN}\left(k_{tar},l_{tar}\right)$ can be expressed as

(29) $$P_{SN}\left(k_{tar},l_{tar}\right)=\left\{\begin{array}{c} 1,\;j={\left[k-kp\right]}_{N}M+{\left[l-lp\right]}_{M}\\ 0,\;otherwise \end{array}\right.$$

where i=kM+l. Let $Q_{SN}\left(k_{tar},l_{tar}\right)=\Lambda_{SN}\left(k_{tar},l_{tar}\right)P_{SN}\left(k_{tar},l_{tar}\right)$, (28) can be expressed as

(30) $$y_{SN}=\sqrt{P_{t}}\alpha N_{t}Q_{SN}\left(k_{tar},l_{tar}\right)x_{SM}+w_{SN},$$

We construct a matrix $\Psi \in C^{NM\times NM}$. For the *i*-th row of Ψ where i=kM+l, $\Psi \left(i,:\right)={\left(\frac{1}{\sqrt{P_{t}}N_{t}P_{SM}}Q_{SN}\left(k,l\right)x_{SM}\right)}^{H}$, $P_{SM}=x_{SM}^{H}x_{SM}$ is the power of transmitting signal. Then, the output $\xi_{SN}\in C^{NM\times 1}$ of the proposed MF algorithm can be expressed as

(31) $$\xi_{SN}=\Psi y_{SN}.$$

Peaks will appear in the indices corresponding to $k_{tar}$ and $l_{tar}$ in $|\xi_{SN}|$; hence, target presence can be detected through the constant false alarm rate (CFAR) algorithm. Additionally, by utilizing i=kM+l, the delay and Doppler frequency of the target can be estimated. Specifically, if the $\hat{i}$-th element of $|\xi_{SN}|$ is a peak, then the corresponding estimated delay tap ${\hat{l}}_{tar}$, Doppler tap ${\hat{k}}_{tar}$, and TSC $\hat{\alpha }$ are

(32) $${\hat{l}}_{tar}={\left[\hat{i}\right]}_{M},$$

(33) $${\hat{l}}_{tar}=\left\lfloor \frac{\hat{i}}{M}\right\rfloor ,$$

(34) $$\hat{\alpha }=\xi_{SN}\left[\hat{i}\right],$$

where $\left\lfloor •\right\rfloor$ is the floor operation. Note that Ψ can be precomputed offline. The CP-OTFS MF-based target detection and parameter estimation (MF-DaPE) algorithm is summarized in Algorithm 1.
 **Algorithm 1:** The CP-OTFS MF-DaPE algorithm  **Input**: Ψ, $y_{SN}$  **Output**: ${\hat{l}}_{tar}$, ${\hat{k}}_{tar}$, $\hat{\alpha }$  _1_  Calculate $\xi_{SN}$ through (31);  _2_  Find peak index in $|\xi_{SN}|$ through CFAR algorithm;  _3_  Calculate ${\hat{l}}_{tar}$ through (32);  _4_  Calculate ${\hat{k}}_{tar}$ through (33);  _5_  Calculate $\hat{\alpha }$ through (34);  _6_  Return ${\hat{l}}_{tar}$, ${\hat{k}}_{tar}$ and $\hat{\alpha }$.

#### 2.2.2. The JWM of ISWSCPTS

In the JWM, the ISWSCPTS accomplishes target tracking, communication, and power transfer. In this work mode, assuming an approximate target location and using the channel state information (CSI) of the CN and ERN as priors is reasonable, as target detection has already been achieved in the SM, and CSI can be obtained by transmitting pilot signals. The ISWSCPTS aims to achieve better performance in the JWM. In this work, we enhance perception performance while ensuring communication and power transfer performance by designing the transmitting CP-OTFS signal and beamforming.

*A. The receiving model of JWM*

The DD domain-received echo of the target in TM can be expressed as

(35) $$z_{SN}=\sqrt{P_{t}}\alpha a_{t}^{T}\left(\theta_{tar}\right)\eta Q_{SN}\left(k_{tar},l_{tar}\right)x_{JWM}+w_{SN},$$

Similarly, the DD domain-received signal in CN can be expressed as follows:

(36) $$z_{CN}=\sqrt{P_{t}}\beta a_{t}^{T}\left(\theta_{CN}\right)\eta Q\left(k_{CN},l_{CN}\right)x_{JWM}+w_{CN},$$

where β is the complex channel gain, $a_{t}\left(\theta_{IR}\right)\in C^{N_{t}\times 1}$ is the steering vector corresponding to angle of CN, $Q_{CN}$ is the communication channel response matrix with delay tap $l_{CN}$ and Doppler tap $k_{CN}$, $w_{CN}\in C^{NM\times 1}$ is the AWGN matrix with the variance of each entry being $\sigma_{CN}^{2}$.

The DD domain-received signal in ERN can be expressed as follows:

(37) $$z_{ERN}=\sqrt{P_{t}}\gamma a_{t}^{T}\left(\theta_{ERN}\right)\eta Q\left(k_{ERN},l_{ERN}\right)x_{JWM}+w_{ERN},$$

where γ is the power transfer channel gain, $a_{t}\left(\theta_{ERN}\right)\in C^{N_{t}\times 1}$ is the transmitting steering vector, $Q_{ERN}$ is the communication channel response matrix with delay tap $l_{ERN}$ and Doppler tap $k_{ERN}$, $w_{ERN}\in C^{NM\times 1}$ is the AWGN matrix with the variance of each entry being $\sigma_{ERN}^{2}$.

*B. Waveform design by ambiguity function shaping*

The ambiguity function (AF) is crucial metrics for assessing radar signals. To enhance the target tracking performance of the system, we formulate an optimization problem aimed at reshaping the AF to achieve lower integral side-lobe levels (ISLs). The definition of the traditional radar signal ambiguity function is as follows [16]:

(38) $$A\left(\tau ,f_{d}\right)=\int x\left(t\right)x^{*}\left(t-\tau \right)\exp\left(j2\pi f_{d}t\right)dt,$$

where x(t) is the transmitting signal, τ is the delay, and $f_{d}$ is the Doppler frequency. However, the output signal of the OTFS system belongs to the delay-Doppler (DD) domain, and the traditional time domain approach cannot be used to define the OTFS ambiguity function. For OTFS, a discrete AF in the DD domain has been proposed, with the expression as follows:

(39) $$A_{k_{0},l_{0}}\left[k,l\right]={\left|x^{H}Q\left(k,l\right)Q\left(k_{0},l_{0}\right)x\right|}^{2},$$

where x is the DD domain transmitting signal, and $l_{0}$ and $k_{0}$ are the delay tap and Doppler tap of interest, respectively. The ISL of $A_{k_{0},l_{0}}\left[k,l\right]$ is

(40) $$\mathrm{ISL}=\sum_{k=0,k\neq k_{0}}^{N-1}\sum_{l=0,l\neq l_{0}}^{M-1}{\left|x^{H}Q\left(k,l\right)Q\left(k_{0},l_{0}\right)x\right|}^{2},$$

Thus, the problem of waveform design for AF shaping is expressed as

(41) $$P_{0}:\;\begin{array}{cc} \min_{x_{JWM}}\; & \sum_{k=0,k\neq k_{0}}^{N-1}\sum_{l=0,l\neq l_{0}}^{M-1}{\left|x_{JWM}^{H}Q\left(k,l\right)Q\left(k_{0},l_{0}\right)x_{JWM}\right|}^{2}\\ s.t.\; & |x_{JWM}\left(i\right)|=1,i=1,2,\ldots ,NM \end{array}$$

The optimization objective in (41) represents the sum of the energy of all mismatched delay-Doppler pairs in the AF. The goal of minimizing this objective is to make the output of the MF for detection as small as possible to reduce false alarms while minimizing interference introduced by uninterested delay and Doppler regions. The constraint of (41) is a constant modulus constraint, which is preferred by radar system. Based on proposition 1 presented in [17], $P_{0}$ can be handled by sequentially solving the following approximation problem:

(42) $$P_{t+1}:\;\begin{array}{cc} \max_{x_{JWM}}\; & u(x_{JWM},x_{JWM}^{\left(t\right)})\\ s.t.\; & |x_{JWM}\left(i\right)|=1,i=1,2,\ldots ,NM \end{array}$$

where $x_{JWM}^{\left(t\right)}$ denotes the *t*-th iteration solution of the proposed iteration algorithm. The expression of $u(x_{JWM},x_{JWM}^{\left(t\right)})$ is

(43) $$u\left(x_{JWM},x_{JWM}^{\left(t\right)}\right)=x_{JWM}^{H}\left(\lambda^{\left(t\right)}I_{NM}-\Theta \left(x_{JWM}^{\left(t\right)}\right)\right)x_{JWM},$$

where $\lambda^{\left(t\right)}$ satisfies that $\lambda^{\left(t\right)}\geq \beta \lambda_{\max}\left(\Theta \left(x_{JWM}^{\left(t\right)}\right)\right)$. Let $\Gamma_{k_{0},l_{0}}\left(k,l\right)=Q\left(k,l\right)Q\left(k_{0},l_{0}\right)$, and Θ(x) is expressed as

(44) $$\Theta \left(x\right)=\frac{1}{2(NM-1)}\sum_{k=0,k\neq k_{0}}^{N-1}\sum_{l=0,l\neq l_{0}}^{M-1}\left(\Gamma_{k_{0},l_{0}}{\left(k,l\right)}^{H}xx^{H}\Gamma_{k_{0},l_{0}}\left(k,l\right)+\Gamma_{k_{0},l_{0}}\left(k,l\right)xx^{H}\Gamma_{k_{0},l_{0}}{\left(k,l\right)}^{H}\right).$$

Furthermore, according to proposition 2 in [17], $P_{t+1}$ can be solved through the following problem:

(45) P1:maxx^JWMRe(x^JWMHv(t))s.t.|x^JWM(i)|=1,i=1,2,…,NM

where $v^{\left(t\right)}$ can be expressed as

(46) $$v^{\left(t\right)}=\left(\lambda^{\left(t\right)}I_{NM}-\Theta \left(x_{JWM}^{\left(t\right)}\right)\right)x_{JWM}^{\left(t\right)}.$$

Then, the closed-form solution for $P_{1}$ is

(47) $${\hat{x}}_{JWM}=\exp\left(j\arg\left(v^{\left(t\right)}\right)\right),$$

where exp(•) and arg(•) are applied element-wise to the vectors.

Note that the optimal waveform $x_{JWM}^{★}$ can also be utilized in SM. This waveform design algorithm is called CP-OTFS AFS, and it is summarized in Algorithm 2.
 **Algorithm 2:** CP-OTFS AFS  **Input**: Initial $x_{JWM}^{\left(0\right)}$ and stop condition ϵ  **Output**: The optimized waveform $x_{JWM}^{★}$  _1_  Let t←0 and $x_{JWM}^{\left(t\right)}\leftarrow x_{JWM}^{\left(0\right)}$;  _2_  $v^{\left(t\right)}\leftarrow \left(\lambda^{\left(t\right)}I_{NM}-\Theta \left(x_{JWM}^{\left(t\right)}\right)\right)x_{JWM}^{\left(t\right)}$;  _3_  ${\hat{x}}_{JWM}\leftarrow \exp\left(j\arg\left(v^{\left(t\right)}\right)\right)$;  _4_  t←t+1;  _5_  $x_{JWM}^{\left(t\right)}\leftarrow {\hat{x}}_{JWM}$;  _6_  If $|x_{JWM}^{\left(t\right)}-x_{JWM}^{\left(t-1\right)}|\leq \epsilon$, return $x_{JWM}^{★}=x_{JWM}^{\left(t\right)}$. Otherwise, return to step 2.

Now, we analyze the time complexity and space complexity of CP-OTFS AFS. For each iteration, the computational focus of CP-OTFS AFS is on calculating Θ, as shown in (44). Therefore, we approximate the time complexity of CP-OTFS AFS based on the time complexity of calculating Θ. It is noted that $\Gamma_{k_{0},l_{0}}\left(k,l\right)$, used in the computation of Θ, does not need to be recalculated during iterations; hence, the computation primarily centers around the matrix multiplication in (44). Through analysis, the time complexity of CP-OTFS AFS is approximately $O(\left(N-1\right)\left(M-1\right)\left[4{\left(NM\right)}^{3}+{\left(NM\right)}^{2}\right])$.

In each iteration of CP-OTFS AFS, the required storage space includes variables $x_{JWM}^{\left(t\right)}$, $x_{JWM}^{H}$, Θ, $\Gamma_{k_{0},l_{0}}\left(k,l\right)$, $\Gamma_{k_{0},l_{0}}{\left(k,l\right)}^{H}$, $I_{NM}$, and $v^{\left(t\right)}$. Therefore, the space complexity of CP-OTFS AFS is $O(\left(2\left(N-1\right)\left(M-1\right)+2\right){\left(NM\right)}^{2}+3NM)$.

*C. Beamforming Design for ISWSCPTS*

In this section, we investigate the beamforming design for ISWSCPTS. Within the beamforming design challenges of ISWSCPTS, two key considerations emerge: (1) the optimization of sensing performance; (2) fulfilling the requirements for power transfer in the context of ERN considerations.

We begin by deriving metrics for sensing, communication, and power transfer. Subsequently, we formulate the optimization problem for the ISWSCPTS beamforming design. Finally, we propose an algorithm to solve the beamforming design optimization problem.

*C1. Sensing metric*

In the JWM, the ISWSCPTS necessitates the continuous estimation of target parameters, and the precision of target parameter estimation is closely tied to the signal-to-noise ratio (SNR). Consequently, we employ the SNR as the sensing metric.

According to (25), the ${\mathrm{SNR}}_{\mathrm{SN}}$ is

(48) $$\begin{array}{cc} {\mathrm{SNR}}_{\mathrm{SN}} & =\frac{E\{||\sqrt{P_{t}}\alpha a_{t}^{H}\left(\theta_{tar}\right)\eta \left(h_{SN}⊙x_{SN}\right)|{|}_{F}^{2}\}}{E\{||w_{SN}|{|}_{F}^{2}\}}. \end{array}$$

Based on the properties of $|h_{SN}{\left(j\right)|}^{2}=1$ and $|x_{SN}{\left(j\right)|}^{2}=1$, through a series of calculations, (48) can be simplified to

(49) $$\begin{array}{cc} {\mathrm{SNR}}_{\mathrm{SN}} & =\frac{P_{t}{\left|\alpha \right|}^{2}\eta^{H}a_{r}\left(\theta_{tar}\right)a_{r}^{H}\left(\theta_{tar}\right)\eta }{\sigma_{SN}^{2}}\\ \; & =\eta^{T}G_{\mathrm{SN}}\eta . \end{array}$$

where $G_{\mathrm{SN}}=\frac{P_{t}{\left|\alpha \right|}^{2}}{\sigma_{SN}^{2}}a_{r}\left(\theta_{tar}\right)a_{r}^{H}\left(\theta_{tar}\right)$.

*C2. Communication metric*

Due to the impact of communication quality, such as the bit error rate (BER) and channel capacity, being closely related to SNR and in order to maintain consistency with sensing metrics, the SNR is also employed as the communication metric. Similar to derivation of sensing metric, the ${\mathrm{SNR}}_{\mathrm{CN}}$ is

(50) $$\begin{array}{cc} {\mathrm{SNR}}_{\mathrm{CN}} & =\frac{E\{||\sqrt{P_{t}}\beta a_{t}^{H}\left(\theta_{CN}\right)\eta {\left(h_{CN}⊙x_{CN}\right)}^{T}|{|}_{F}^{2}\}}{E\{||w_{CN}|{|}_{F}^{2}\}}\\ \; & =\frac{P_{t}{\left|\beta \right|}^{2}\eta^{H}a_{t}\left(\theta_{CN}\right)a_{t}^{H}\left(\theta_{CN}\right)\eta }{\sigma_{CN}^{2}}\\ \; & =\eta^{H}G_{\mathrm{CN}}\eta , \end{array}$$

where $G_{\mathrm{CN}}=\frac{P_{t}{\left|\beta \right|}^{2}}{\sigma_{CN}^{2}}a_{t}\left(\theta_{CN}\right)a_{t}^{H}\left(\theta_{CN}\right)$.

*C3. Power transfer metric*

The ERN collects energy from signals emitted by the SN; thus, the harvested energy is employed as the metric of power transfer. Due to the negligible power of noise compared with transmitting signal, the harvested energy can be expressed as

(51) $$\begin{array}{cc} E_{\mathrm{ERN}} & =E\{\mu ||\sqrt{P_{t}}\gamma a_{t}^{H}\left(\theta_{ERN}\right)\eta {\left(h_{ERN}⊙x_{ERN}\right)}^{T}|{|}_{F}^{2}\}\\ \; & =\mu P_{t}{\left|\gamma \right|}^{2}\eta^{H}a_{r}\left(\theta_{ERN}\right)a_{r}^{H}\left(\theta_{ERN}\right)\eta \\ \; & =\eta^{H}G_{\mathrm{ERN}}\eta , \end{array}$$

where μ∈[0,1] is the energy-harvesting efficiency, $G_{\mathrm{CN}}=\mu P_{t}{\left|\gamma \right|}^{2}a_{r}\left(\theta_{ERN}\right)a_{r}^{H}\left(\theta_{ERN}\right)$.

*C4. SDR-BD algorithm*

As we want to optimize the sensing performance of ISWSCPTS while meeting the basic requirements for communication and power transfer, the problem of the beamforming design can be expressed as

(52) $$P_{2}:\;\begin{array}{cc} \max_{\eta }\; & \eta^{H}G_{\mathrm{SN}}\eta \\ s.t.\; & \eta^{H}G_{\mathrm{CN}}\eta^{\geq }\rho \\ \; & \eta^{H}G_{\mathrm{ERN}}\eta \geq \varrho \\ \; & \eta^{H}\eta \leq 1 \end{array}$$

where ρ represents the minimum SNR required for communication, and ϱ denotes the minimum energy requirement for triggering the energy-harvesting process in ERN.

The optimization objective of $P_{2}$ is the received SNR of the SN. By optimizing the SNR of the SN, we aim to maximize sensing performance under the QoS constraints of communication and PT, since the detection and parameter estimation performance of the SN improves with an increasing SNR. This optimization objective is related to the target position and η. Since the JWM of the ISWSCPTS has target position information, η can be optimized to maximize the SNR.

Due to the max operation and quadratic constraints, problem $P_{2}$ is a non-convex optimization. In this work, $P_{2}$ is solved through the SDR technique [18]. According to $x^{H}Ax=\mathrm{Tr}\left(Axx^{H}\right)$, where Tr(•) denotes the trace operation, $P_{2}$ can be transformed into the following equivalent optimization problem:

(53) $$P_{3}:\;\begin{array}{cc} \max_{\Gamma }\; & \mathrm{Tr}(G_{\mathrm{SN}}\Gamma )\\ s.t.\; & \mathrm{Tr}(G_{\mathrm{CN}}\Gamma )\geq \rho \\ \; & \mathrm{Tr}(G_{\mathrm{ERN}}\Gamma )\geq \varrho \\ \; & \mathrm{Tr}(\Gamma )\leq 1\\ \; & \mathrm{rank}(\Gamma )=1\\ \; & \Gamma ⪰(0) \end{array}$$

where $\Gamma =\eta \eta^{H}$.

Then, the rank constraint is dropped to obtain the following relaxed optimization problem:

(54) $$P_{4}:\;\begin{array}{cc} \max_{\Gamma }\; & \mathrm{Tr}(G_{\mathrm{SN}}\Gamma )\\ s.t.\; & \mathrm{Tr}(G_{\mathrm{CN}}\Gamma )\geq \rho \\ \; & \mathrm{Tr}(G_{\mathrm{ERN}}\Gamma )\geq \varrho \\ \; & \mathrm{Tr}(\Gamma )\leq 1\\ \; & \Gamma ⪰(0) \end{array}$$

The objective function and constraints in $P_{4}$ are all affine, thus making $P_{4}$ a convex optimization problem. $P_{4}$ can be solved by Matlab using the convex optimization toolbox CVX. If the rank of the solution $\Gamma^{★}$ obtained in $P_{4}$ is not equal to 1, then feasible solutions $\eta^{★}$ need to be extracted from $\Gamma^{★}$. Following [18], $\eta^{★}$ can be obtained as follows:

(55) $$\eta^{★}=\sqrt{\lambda_{0}}q_{0}$$

where $\lambda_{0}$ represents the maximum eigenvalue of $\Gamma^{★}$, and $q_{0}$ denotes the corresponding eigenvector.

Note that the SDR-BD algorithm is a quadratic programming problem. According to [19], the time complexity of quadratic programming problems is $O(N^{3})$, and the space complexity is $O(N^{2})$. Therefore, the time complexity of SDR-BD is $O({\left(NM\right)}^{3})$, and the space complexity is $O({\left(NM\right)}^{2})$.

## 3. Results and Discussions

This section conducts simulations to evaluate the performance of CP-OTFS MF-TDaPE, CP-OTFS AFS, and SDR-BD. The basic simulation parameters are summarized in Table 2.

### 3.1. Simulation of CP-OTFS MF-TDaPE

**Figure 3:**
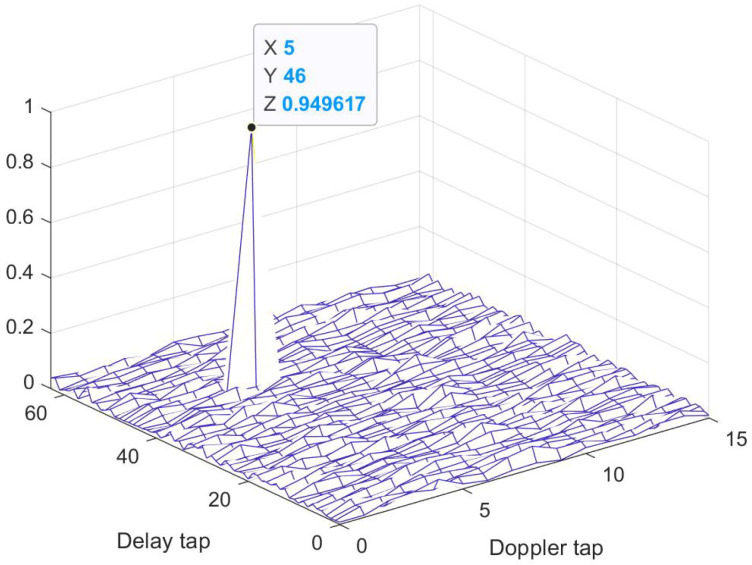
The output of MF.

To begin with, target detection is performed using the CP-OTFS MF-TDaPE algorithm. The target range and velocity are 689.523 m and 95.054 m/s, corresponding to $l_{p}=46$ and $k_{p}=5$, respectively, with a TSC of 0.8247+0.4709i. The SNR of this simulation is 10 dB. Figure 3 illustrates the output $\xi_{SN}$ of the MF, where a significant peak appears at positions l=46 and k=5. The target can be correctly detected using the CFAR algorithm.

To validate the parameter estimation performance of the CP-OTFS MF-TDaPE, we conduct parameter estimation for fast-moving targets with different velocities. The benchmark is the OFDM waveform, and the estimation method is the fast Fourier transform (FFT) method described in [20]. Since both CP-OTFS and OFDM can accurately estimate the range, the main comparison focuses on the velocity estimation performance and TSC estimation performance. The root mean square error (RMSE) is adopted as the performance metric and is calculated as follows:

(56) $$RMSE={\left(\frac{\sum_{n=1}^{Ns}{\left|\hat{y}-y\right|}^{2}}{N_{s}}\right)}^{\frac{1}{2}}$$

where $\hat{y}$ is the estimated parameter, *y* is the true value, and $N_{s}$ is the number of samples.

**Figure 4:**
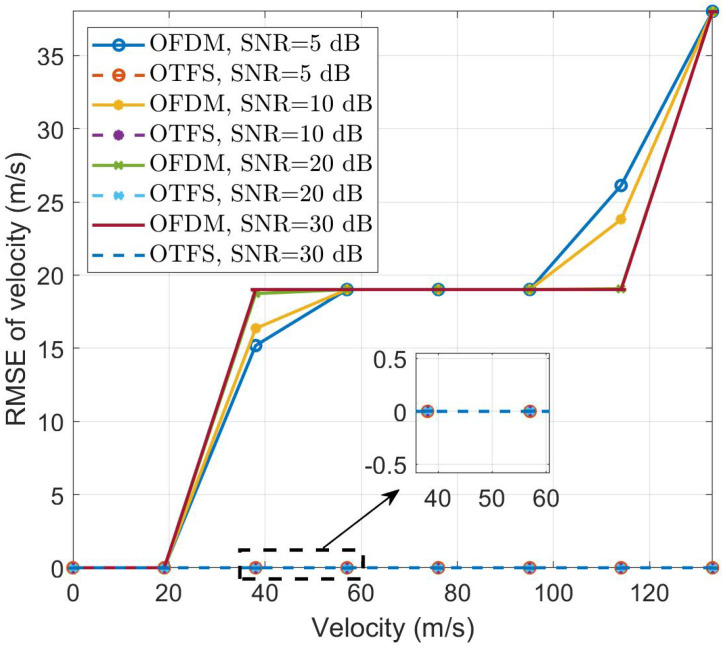
Velocity RMSE vs. relative velocity.

**Figure 5:**
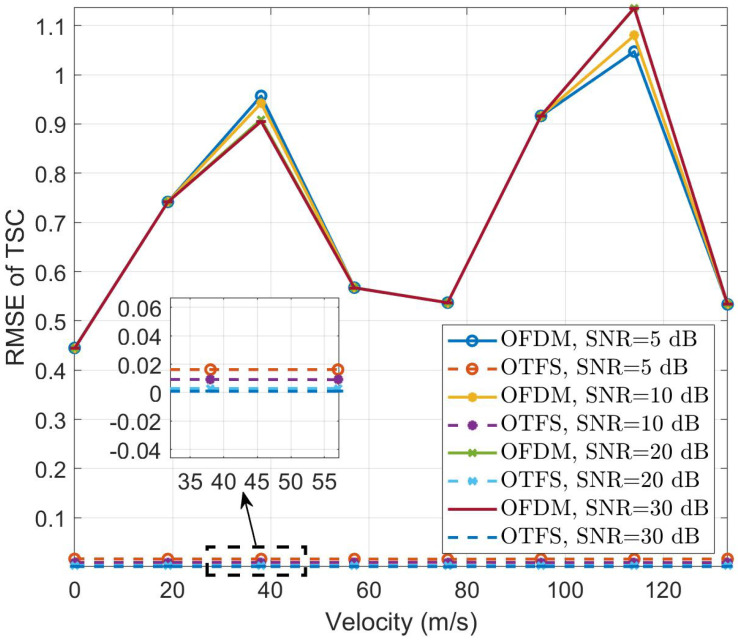
TSC RMSE vs. relative velocity.

Figure 4 and Figure 5 depict the RMSE results for the velocity and TSC estimation of fast-moving targets with different velocities, respectively. In the experiments for Figure 4 and Figure 5, the SNRs are set to 5 dB, 10 dB, 20 dB, and 30 dB, and the target range is 689.523 m, corresponding to a delay tap of 46. The target’s TSC is set to 0.8247+0.4709i. We then set the target velocities to [0 m/s, 19.01 m/s, 38.02 m/s, 57.03 m/s, 76.04 m/s, 95.05 m/s, 114.06 m/s, 133.07 m/s], corresponding to Doppler taps of [0, 1, 2, 3, 4, 5, 6, 7]. It is evident that for fast-moving targets, the performance of velocity estimation sharply deteriorates with increasing velocity in OFDM, while OTFS consistently maintains accurate estimation. Regarding TSC estimation, although OFDM does not exhibit sensitivity to velocity variations, its estimation results still pale in comparison to the nearly error-free estimates provided by CP-OTFS. The results presented demonstrate the robustness of the ISWSCPTS framework in parameter estimation for high-speed motion scenarios.

Interestingly, in Figure 4, when the target’s relative velocity is low, such as 38.02 m/s, OFDM performs better at a low SNR. Conversely, at high velocities, OTFS outperforms OFDM. This phenomenon can be explained by OFDM’s weak anti-Doppler capability, causing a “misalignment” of the peak used for parameter estimation. At low velocities, this “misalignment” is small. At a low SNR, the noise can “correct” this misalignment. However, at high velocities, the “misalignment” becomes significant, and the noise’s “correcting” effect becomes negligible.

The phenomenon observed in Figure 5 is also interesting. For OFDM, at low velocities, such as 38.02 m/s, the TSC estimation performance is intuitive. However, at high velocities, such as 114.06 m/s, the results at a high SNR are unexpectedly worse. This is because, at low velocities, the primary source of TSC estimation error is noise, whereas at high velocities, it is Doppler spread. Additionally, the previously mentioned “correcting” effect of noise at a low SNR also plays a role.

The explanation for the near-perfect CP-OTFS results in Figure 4 and Figure 5 is as follows: In CP-OTFS systems, the DDC model induces sparsity in the channel and a low correlation between time and frequency domains, resulting in discrete channel impulses in the DD domain. Due to these characteristics, CP-OTFS systems can achieve perfect Doppler estimation performance. In contrast, OFDM experiences strong ICI in high-speed scenarios, leading to significant performance degradation.

### 3.2. Simulation of CP-OTFS AFS

**Figure 6:**
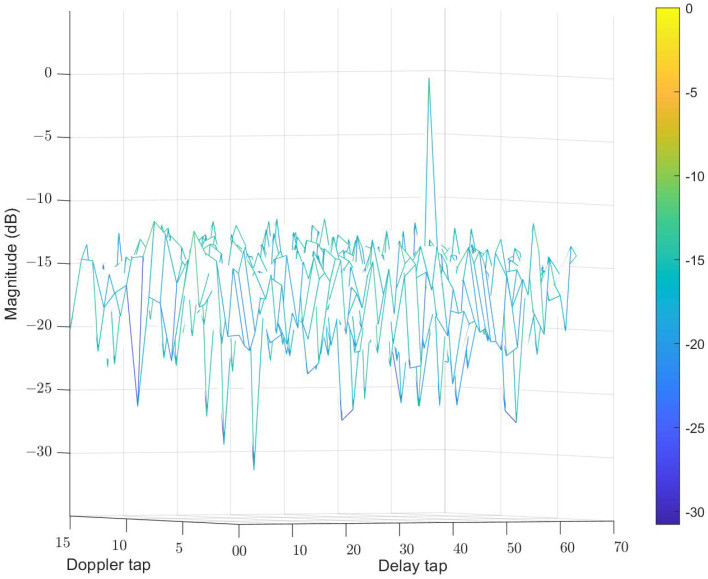
AF of initial waveform. The different colors in the picture represent different magnitudes.

**Figure 7:**
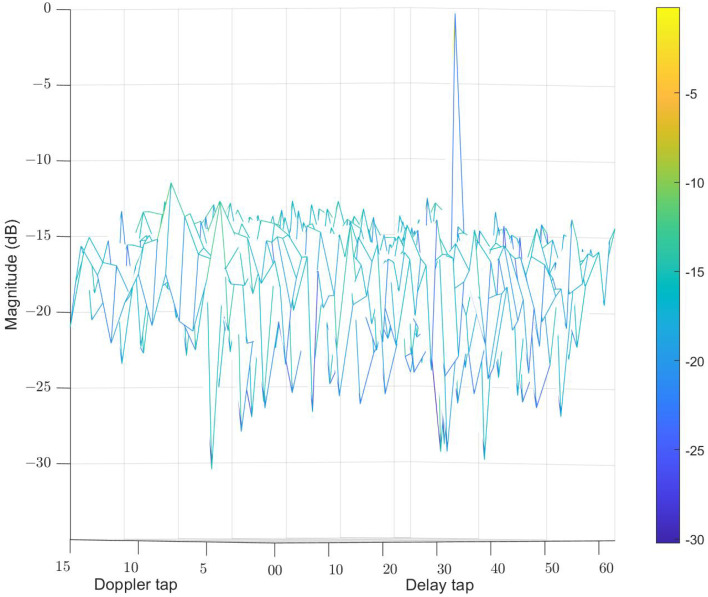
AF of optimized waveform. The different colors in the picture represent different magnitudes.

The initial waveform symbols for this simulation are quadrature phase shift keying (QPSK) symbols. We first compare the AF of the waveform optimized by CP-OTFS AFS with the initial waveform. Figure 6 and Figure 7, respectively, depict the AF of the initial and optimized waveforms. In the experiments for Figure 6 and Figure 7, the initial waveform is set to a random QPSK signal, with the interested delay tap and Doppler tap set to 46 and 5, respectively. Using the initial and optimized signals, the corresponding AFs are obtained according to (39). It can be observed that the optimized waveform exhibits a reduction of approximately 2–3 dB in side lobes in comparison to the initial waveform.

**Figure 8:**
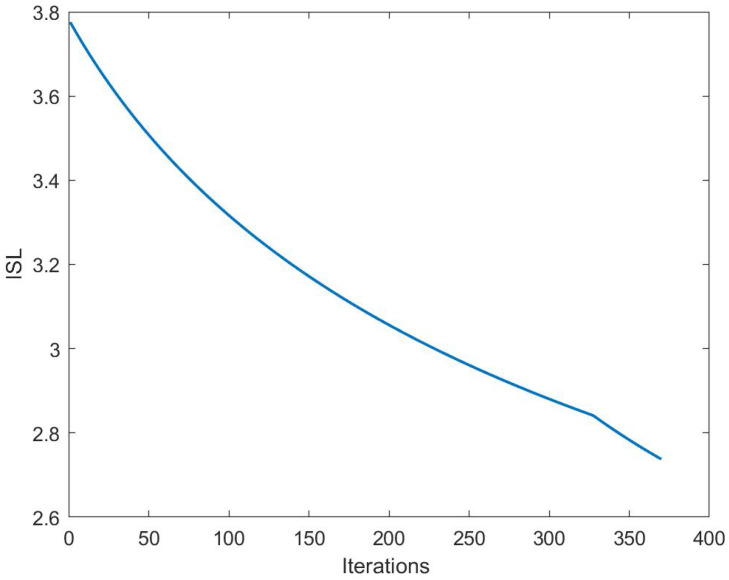
ISL vs. iterations.

Figure 8 illustrates the variation in the ISL with the number of iterations, showing a gradual decrease with successive iterations of the algorithm. However, the convergence speed of CP-OTFS AFS is slow, and there is still potential for improvement.

**Figure 9:**
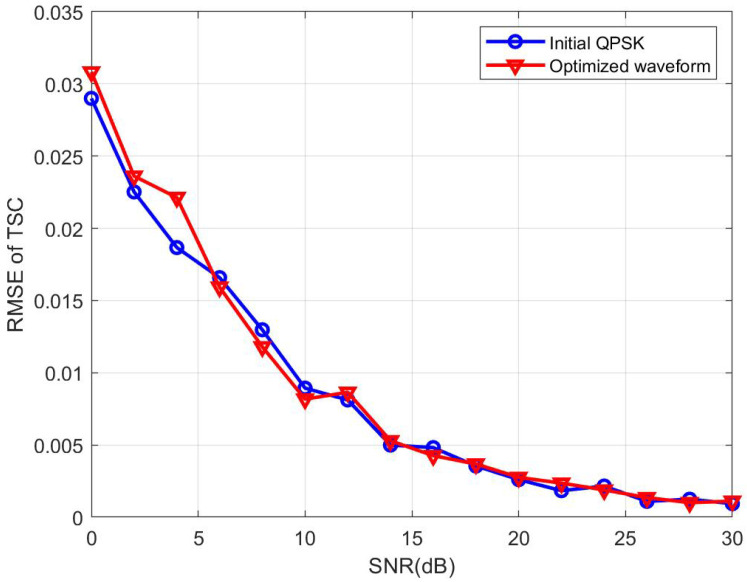
TSC RMSE vs. SNR under interference-free conditions.

In fact, the performance of CP-OTFS MF-TDaPE in estimating TSC deteriorates with the emergence of interference. To illustrate this point, we introduced interference targets in the simulation scenario. In the experiment for Figure 9, there are no interference targets, the target’s delay tap and Doppler tap are set to 46 and 5, respectively, and the target’s TSC is set to 0.8247+0.4709i. The MF-TDaPE algorithm is then used to estimate the target’s TSC at different SNRs, and the corresponding RMSE is calculated, completing the comparative experiment. Figure 9 shows the variation in the target TSC with the SNR in the absence of interference, indicating similar performance between the initial QPSK waveform and the optimized waveform.

**Figure 10:**
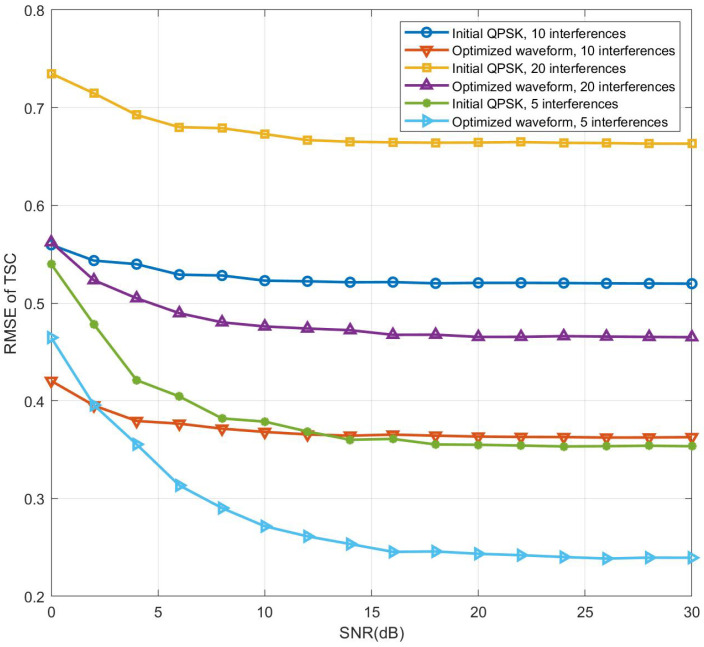
TSC RMSE vs. SNR under interference conditions.

In the experiment for Figure 10, based on the settings of the experiment in Figure 9, several interfering targets are added. The delay taps and Doppler taps of the interfering targets are randomly selected from [0,M−1] and [0,N−1], respectively, and the TSCs are complex Gaussian random variables with a mean of 0 and a variance of 1. The MF-TDaPE algorithm is then used to estimate the targets’ TSCs at different SNRs, and the corresponding RMSE is calculated, completing the comparative experiment. Figure 10 illustrates the TSC estimation performance in the presence of interference. It is evident that the performance is degraded. The reason is that the interference target affects TSC estimation through AF sidelobes. At the same time, it can also be observed that the performance of the algorithm deteriorates as the number of interfering targets increases.

### 3.3. Simulation of SDR-BD

This simulation showcases the beam patterns designed by SDR-BD under different system configurations and QoS settings. The specific simulation parameters for this study are detailed in Table 3.

**Figure 11:**
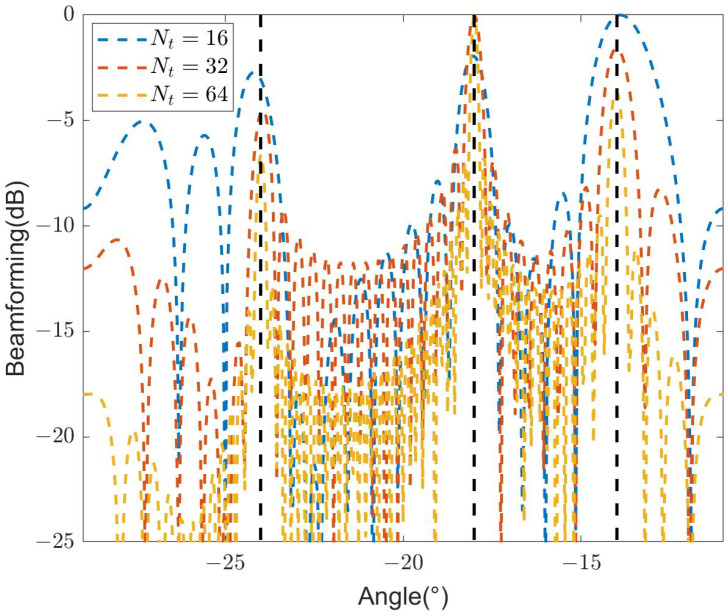
Designed beamforming of different $N_{t}$.

Figure 11 illustrates the beamforming design results of SDR-BD under different configurations of $N_{t}$. The simulation result demonstrates that as $N_{t}$ increases, the beamforming becomes narrower in the directions of the target, CN, and ERN, indicating stronger directivity. Additionally, the beamforming exhibits lower sidelobes. Particularly, the highest sidelobe level of the beamforming near the target with $N_{t}=64$ is approximately 6.5 dB lower than that with $N_{t}=16$. This phenomenon arises from the increase in system antenna aperture with $N_{t}$, resulting in an improved angular resolution of the system.

**Figure 12:**
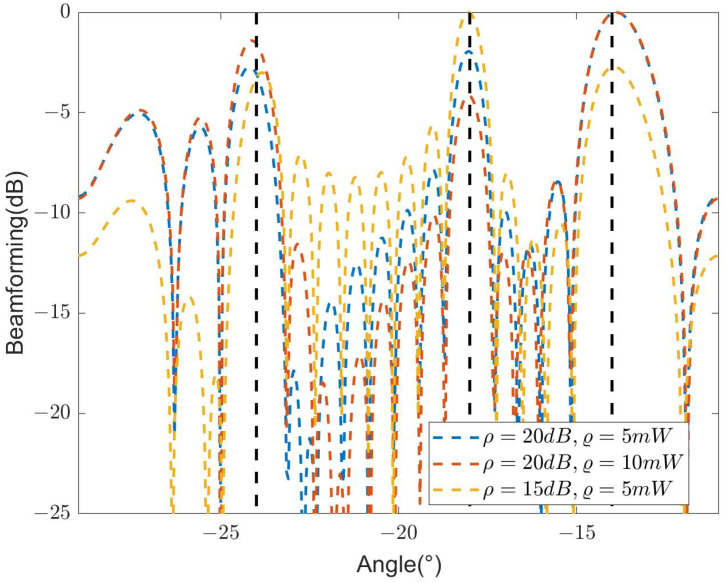
Designed beamforming of different QoS settings.

Figure 12 compares the beamforming with $N_{t}=16$ designed by SDR-BD under different communication and PT QoS settings. The simulation result indicates that increasing the QoS for communication and PT inevitably leads to a deterioration in sensing capability. Specifically, compared to the case where ρ = 15 dB and ϱ = 5 mW, an increase of 5 dB in ρ and 5 mW in ϱ results in a reduction of approximately 4.8 dB in the antenna gain in the direction of the target. This outcome suggests that the beamforming designed by SDR-BD achieves a trade-off among sensing, communication, and PT in ISWSCPTS. Therefore, in practical application scenarios, the overall system performance optimization can be achieved according to the respective QoS requirements.

## 4. Conclusions

In this work, the ISWSCPTS framework, which integrates ISACS, SWIPT, and OTFS, is proposed. ISWSCPTS comprises two work modes, SM and JWM, which utilize a single CP-OTFS waveform. SM is employed for target detection and parameter estimation, while JWM is used for target tracking, communication, and PT. For target detection and parameter estimation, the CP-OTFS MF-DaPE algorithm is proposed, demonstrating superior performance in high-speed scenarios compared to OFDM. To enhance the robustness of parameter estimation against interference, the CP-OTFS AFS algorithm is employed for waveform design. The waveform designed by CP-OTFS AFS reduces the ISL of the corresponding AF and improves the interference resilience. The SDR-BD algorithm is proposed to enhance the overall perceptual capability of the system by designing beamforming to maximize perceptual capability under communication and PT QoS constraints.

However, this work still has the following limitations: (1) the ISWSCPTS scenarios include only a single target, CN, and ERN; (2) CP-OTFS AFS incurs a significant computational burden. In future work, we will focus on extending the ISWSCPTS to multiple target, CN, and ERN scenarios and optimizing CP-OTFS AFS to improve its convergence speed.

## Figures and Tables

**Table 1:** Shortcomings of existing technologies.

Existing Technologies	Shortcomings
ISACS	Lacking expansion on PT.
OFDM	Introducing severe ICI In high-speed scenarios.
OTFS-based radar in [9]	Inaccuracy in estimating TSC.
System in [15]	Lacking consideration for high-speed scenarios.

**Table 2:** Basic simulation parameters.

Symbol	Parameter	Value
$f_{c}$	Carrier frequency	77 GHz
*N*	Number of Doppler samples	16
*M*	Number of delay samples	64
*B*	Total bandwidth	10 MHz
Δf	Subcarrier spacing	156.250 kHz
ΔR	Range resolution	14.99 m
ΔV	Velocity resolution	19.01 m/s
$R_{max}$	Unambiguous range	959.336 m
$V_{max}$	Unambiguous velocity	±152.086 m/s

**Table 3:** Specific simulation parameters for SDR-BD.

Symbol	Parameter	Value
Pt	Transmitting power	1 W
$\sigma_{CN}^{2}$	Variance of noise in CN	0 dBm
$\sigma_{SN}^{2}$	Variance of noise in SN	10 dBm
α	TSC	0.8247+0.4709i
β	Communication channel gain	0.01
γ	PT channel gain	0.02 m
μ	Energy-harvesting efficiency	0.1
ρ	SNR required in CN	Configured
ϱ	Energy requirement in SN	Configured

## Data Availability

The original contributions presented in the study are included in the article material; further inquiries can be directed to the corresponding author.

## Abbreviations

The following abbreviations are used in this manuscript:

PT	Power transfer
ISACS	Integrated sensing and communication system
SWIPT	Simultaneous wireless information and power transfer
OTFS	Orthogonal Time Frequency Space
4G	Fourth-generation
5G	Fifth-generation
ICI	Inter-carrier interference (ICI)
RF	Radio frequency
CP	Cyclic prefix
ISWSCPTS	Integrated simultaneous wireless sensing, communication, and power transfer system
MF	Matched filter
AF	Ambiguity function
AFS	AF shaping
MF-TDaPE	MF-based target detection and parameter estimation
QoS	Quality of service
SDR	Semidefinite relaxation
SDR-BD	SDR beamforming design
OFDM	Orthogonal frequency division multiplexing
UAV	Unmanned aerial vehicle
SM	Search mode
JWM	Joint work mode
ISL	Integrated sidelobe level
ULA	Uniform linear array
CN	Communication node
ERN	Energy receiving node
SN	Sensing node
TF	Time–frequency
DD	Doppler-delay
DDC	DD channel
SFFT	Symplectic finite Fourier transform
ISFFT	Inverse SFFT
TSC	Target scattering coefficient
CFAR	Constant false alarm rate
DDC	DD channel
CSI	Channel state information
FFT	Fast Fourier transform
RMSE	Root mean square error
SNR	Signal-to-noise ratio
QPSK	Quadrature phase shift keying
